# Endogenous stem cell mobilization and localized immunosuppression synergistically ameliorate DSS-induced Colitis in mice

**DOI:** 10.1186/s13287-024-03777-2

**Published:** 2024-06-13

**Authors:** Shobha Regmi, Shiva Pathak, Dinesh Chaudhary, Jong Oh Kim, Joo-Won Nam, Hyung-Sik Kim, Hu-Lin Jiang, Dongryeol Ryu, Jong-Hyuk Sung, Simmyung Yook, Jee-Heon Jeong

**Affiliations:** 1 College of Pharmacy, Yeungnam University, Gyeongsan, Gyeongbuk 38541 Republic of Korea; 2 Interventional Radiology Innovation at Stanford, Department of Radiology, School of Medicine, Stanford University, Stanford, CA 94304 USA; 3 Division of Blood and Marrow Transplantation, School of Medicine, Stanford University, Stanford, CA 94305 USA; 4 Department of Precision Medicine, School of Medicine, Sungkyunkwan University, Suwon, 16419 Republic of Korea; 5 Department of Life Science in Dentistry, School of Dentistry, Pusan National University, Yangsan, 50612 Republic of Korea; 6 Dental and Life Science Institute, Pusan National University, Yangsan, 50612 Republic of Korea; 7 State Key Laboratory of Natural Medicines, China Pharmaceutical University, Nanjing, 210009 China; 8 Jiangsu Key Laboratory of Druggability of Biopharmaceuticals, China Pharmaceutical University, Nanjing, 210009 China; 9 Jiangsu Key Laboratory of Drug Discovery for Metabolic Diseases, China Pharmaceutical University, Nanjing, 210009 China; 10 NMPA Key Laboratory for Research and Evaluation of Pharmaceutical Preparations and Excipients, China Pharmaceutical University, Nanjing, 210009 China; 11 Department of Biomedical Science and Engineering, Gwangju Institute of Science and Technology, Gwangju, 61005 Republic of Korea; 12 College of Pharmacy, Yonsei Institute of Pharmaceutical Sciences, Yonsei University, Incheon, 21983 Republic of Korea; 13 Epibiotech Co. Ltd., Incheon, 21983 Republic of Korea; 14 Department of Biopharmaceutical Convergence, Sungkyunkwan University, Suwon, 16419 Republic of Korea; 15 School of Pharmacy, Sungkyunkwan University, Suwon, 16419 Republic of Korea

**Keywords:** AMD-3100, FK506, Thioketal microspheres, Stem cell mobilizing effect, Hematopoietic stem cells, Combination therapy

## Abstract

**Background:**

Stem cell therapy is a promising alternative for inflammatory diseases and tissue injury treatment. Exogenous delivery of mesenchymal stem cells is associated with instant blood-mediated inflammatory reactions, mechanical stress during administration, and replicative senescence or change in phenotype during long-term culture in vitro. In this study, we aimed to mobilize endogenous hematopoietic stem cells (HSCs) using AMD-3100 and provide local immune suppression using FK506, an immunosuppressive drug, for the treatment of inflammatory bowel diseases.

**Methods:**

Reactive oxygen species (ROS)-responsive FK506-loaded thioketal microspheres were prepared by emulsification solvent-evaporation method. Thioketal vehicle based FK506 microspheres and AMD3100 were co-administered into male C57BL6/J mice with dextran sulfate sodium (DSS) induced colitis. The effect of FK506-loaded thioketal microspheres in colitis mice were evaluated using disease severity index, myeloperoxidase activity, histology, flow cytometry, and gene expression by qRT-PCR.

**Results:**

The delivery of AMD-3100 enhanced mobilization of HSCs from the bone marrow into the inflamed colon of mice. Furthermore, targeted oral delivery of FK506 in an inflamed colon inhibited the immune activation in the colon. In the DSS-induced colitis mouse model, the combination of AMD-3100 and FK506-loaded thioketal microspheres ameliorated the disease, decreased immune cell infiltration and activation, and improved body weight, colon length, and epithelial healing process.

**Conclusion:**

This study shows that the significant increase in the percentage of mobilized hematopoietic stem cells in the combination therapy of AMD and oral FK506 microspheres may contribute to a synergistic therapeutic effect. Thus, low-dose local delivery of FK506 combined with AMD3100 could be a promising alternative treatment for inflammatory bowel diseases.

**Supplementary Information:**

The online version contains supplementary material available at 10.1186/s13287-024-03777-2.

## Background

Ulcerative colitis (UC) is a common gastrointestinal condition characterized by mucosal injury affecting the large intestines [1]. The treatment of UC involves 5-aminosalicylic acid, antibiotics, corticosteroids, and anti-TNF-α or anti-CD3 antibody therapy [2]. However, long-term use of these drugs results in neurotoxicity, nephrotoxicity, and opportunistic infections. To minimize the adverse effects of drugs, local or targeted drug delivery systems to the colon have been introduced [3–5]. Physiological clues such as mucus secretion, variation in pH in different segments of the gastrointestinal tract, and a series of microbiome secreting enzymes have been used for targeted drug delivery [6]. However, loss of mucus-secreting goblet cells, altered pH, mangled microbiome, and altered GI transit time during UC affect the effectiveness of colon-targeted drug delivery systems where mucoadhesive polymer, bacterial enzyme-based prodrug, extend-release polymer, and pH-responsive materials have been applied to assist targeted-release of drugs to the colon. In that regard, disease-associated changes could be beneficial for targeted drug delivery to the colon. When reactive oxidative species (ROS)-responsive polymer was used to deliver the payload into the colon, accumulation of the payload in the colon significantly increased during UC [7]. Since only few proportion of the patients respond to the conventional treatment methods [8], alternative strategies for the effective treatment of inflammatory bowel disease (IBD) have been investigated.

Mesenchymal stem cell therapy is an alternative for IBD treatment due to its tissue regenerative and immunomodulatory effects [9]. However, the use of exogenously delivered mesenchymal stem cells (MSCs) is associated with the progression of replicative senescence, increased oxidative stress, loss of extracellular matrix, instant blood-mediated inflammatory reactions, and stress during injection [10, 11]. This compromises the therapeutic outcomes of the exogenous MSC-based therapy. Therefore, new approaches to mobilize the endogenous stem cells at the injury site to accelerate tissue repairment and replacement are required. AMD3100 (AMD) is a Food and Drug Administration (FDA)-approved drug for the mobilization of hematopoietic stem cells (HSCs) from the bone marrow to the inflammation site [12, 13]. Several studies have used stem cell mobilization to enhance tissue repairment, transplantation acceptance, and treatment of inflammatory diseases [14–16]. As T and B cells exhibit C-X-C chemokine receptor type 4 (CXCR4) receptors, leucocyte mobilization also occurs during stem cell mobilization [17]. Furthermore, it might aggravate the inflammation and immune reactions at the injured sites [18, 19]. Thus, the combination of AMD with FK506 not only enhances the stem cell mobilization ability of AMD [20] but also inhibits the inflammation by increasing regulatory immune cell population [21]. Many studies found that AMD therapy in combination with a low-dose immunosuppressive regimen effectively controls inflammation and immune activation [20, 21].

In our previous study, we evaluated the efficacy of FK506-loaded thioketal microspheres (TKMs) in the treatment of UC [7]. In the present study, we aimed to combine locally-delivered FK506 with CXCR4 antagonists to enhance epithelial regeneration by mobilizing endogenous stem cells from bone marrow and inhibiting immune cell infiltration. This study revealed, for the first time, that a significant increase in the percentage of hematopoietic stem cells in the combination therapy of AMD and oral FK506-TKM has a synergistic therapeutic effect. In addition, we have shown that a low dose of FK506 combined with AMD treats inflammatory bowel diseases in murine model.

## Materials and methods

### Preparation and characterization of ROS-responsive microspheres

The thioketal polymer was prepared and characterized as described previously [7]. Then, the emulsification solvent-evaporation method was used to prepare ROS-responsive thioketal microspheres (TKMs) [7]. The freeze-dried TKMs were used for physical characterizations and in vivo applications. Morphological characterization was performed using the scanning electron microscope (SEM, S-4100, Hitachi, Tokyo, Japan), and drug loading capacity of FK506-loaded TKMs (FK506-TKMs) was determined by the High-performance liquid chromatography (HPLC) (Thermo Fisher Inc, Waltham, MA, USA) according to a previous method [22].

The microspheres were treated with 1 mM KO_2_ and 1 mM H_2_O_2_ for 48 h to confirm their ROS-responsive behavior. Next, degradation of FK506-TKMs was evaluated using the SEM, and in vitro release of FK506 from FK506-TKMs was performed in phosphate-buffered saline (PBS) (pH 7.4, 0.1% tween 20) with or without 1 mM H_2_O_2_ and 1 mM KO_2_. FK506 in the release sample was estimated using the HPLC.

### Therapeutic effect of FK506-TKM and AMD3100 in colitis animal model

Eight to ten-week-old male C57BL6/J mice were purchased from Samtako (Seoul, Republic of Korea) and were used in the study according to the animal conduct of Yeungnam University, Republic of Korea (IACUC: YL 2018–028) in compliance with ARRIVE (Animal Research: Reporting of In Vivo Experiments) guidelines 2.0. After acclimatization for 1 week, mice were randomly divided into five groups (n = 7); Control, PBS, AMD, FK506-TKMs, and AMD-FK506-TKMs. Afterwards, colitis was induced by administering 3% w/v dextran-sulfate sodium (DSS, NY, USA) in drinking water for 7 days. The mice received daily gavage of PBS (n = 7), or FK506-TKMs (n = 7) (1 mg/kg/day). The group receiving AMD3100 (AMD, Selleck Chemicals, Houston, TX, USA) received AMD at the dose of 1 mg/kg via subcutaneous injection at 0, 2, and 4 days. Bodyweight decrease, stool consistency, and bleeding events were recorded throughout the study period. Furthermore, the disease severity index (DSI) was calculated as described previously [23]. Each animal was assessed for diarrhea, bleeding, and body weight loss, with scores ranging from 0 to 4 indicating severity. These scores were averaged to calculate the DSI for each animal. The animals were humanely euthanized using CO_2_ inhalation according to institutional guidelines and American Veterinary Association (AVMA) guidelines. After sacrificing mice on day 10 of DSS administration, colon length was measured.

### Myeloperoxidase (MPO) assay

To determine the MPO assay, we applied the colorimetric method using O-dianisidine as a substrate, as described [7, 24]. Briefly, the colon was homogenized in 0.5% w/v hexadecyltrimethylammonium bromide (Sigma-Aldrich, St Louis, MO, USA) and prepared in 50 mM PBS (pH 6). The supernatant was used to observe the absorbance at 460 nm, where a change in absorbance was recorded for 15 min using the SPARK 10 M (TECAN, Untersbergstrasse, Grodig, Austria).

### Hematoxylin & Eosin (H&E) staining

After feces removal from the isolated colon with PBS, the colon was fixed in 4% paraformaldehyde for approximately 24 h. Next, the fixed colon was soaked into 30% w/v sucrose for 24‒48 h, and thin sections were prepared using microtome. Then the tissue sections were rehydrated using a series of concentrations of alcohol, stained with hematoxylin and eosin, and finally dehydrated with ethanol gradient and covered with a coverslip using a mounting solution (Biomed, Foster City, CA. USA). An optical microscope (Nikon Eclipse Ti) was used to capture the images. The pathophysiology scoring based on histology was determined by the extent of infiltration of inflammatory cells, epithelial lining destruction, loss of goblet cells, hyperplasia, and cryptitis of the colon.

### Immune cell isolation

Isolation of lymphocytes was performed from colonic lamina propria following the method described previously [25]. Briefly, the colon was washed with PBS to remove feces completely. Furthermore, to obtain single cells, colons were minced and subjected to collagenase digestion to obtain single cells. After digestion, RPMI with 10% FBS was used to neutralize the enzyme action and centrifuged to get cell pellets. The cell pellets were suspended in RPMI and were filtered using a 40 μm cell strainer. The collected cells were washed with PBS and stained with APC-conjugated anti-CD3 antibody for flow cytometry analysis. Similarly, colon-draining lymph nodes (c-MLN) were isolated following a previously described method [26]. For the determination of Th1 and Th17 differentiation, the lymphocytes obtained from c-MLN were stimulated in vitro with 750 ng/mL ionomycin (Calbiochem), GolgiStop™, and 50 ng/mL phorbol 12-myristate 13-acetate (PMA) (Sigma-Aldrich) for 4 h and the analysis was done through flow cytometry as described previously [7].

### Evaluation of stem cell migration in the *colon*

To verify the migration of stem cells to the colon from bone marrow, the percentage of hematopoietic stem cells derived from bone marrow were evaluated. The washed colon was chopped, and collagenase digestion of the colonic tissue was performed using collagenase (1 mg/mL) and DNAse (10 μg/mL) for 30 min at 37 °C with continuous stirring. Thus, obtained single cells were stained with Sca-1 and CD34 antibodies and the percentage double positive population of Sca-1^+^ and CD34^+^ stem cells was evaluated using FACS.

### Western blotting

Colon tissues were lysed using RIPA-containing halt protease inhibitor cocktail (Thermo Scientific, 78,430). Proteins were quantified using the BCA assay kit and the same amount of proteins was resolved to 12% sodium dodecyl sulfate–polyacrylamide gel electrophoresis (SDS-PAGE), then transferred to an Immobilon-P transfer membrane (Millipore Corporation, Billerica, MA, USA). Then, 5% of skim milk was used for blocking the membrane and separately incubated with primary antibodies against COX-2 (1:1000; Cell Signaling Technology, 12282S), and β-actin (1:1000; Cell Signaling Technology, 4970S) overnight at 4 °C. Further, membranes were incubated with HRP-conjugated secondary antibodies for 1 h at room temperature and images were developed using a chemiluminescence detection kit (Thermo Scientific). The β-actin level was used as a loading control. Expressed protein density was analyzed using GELQuantNET software (BiochemLab Solution, San Francisco, CA, USA).

### Total RNA isolation and quantitative real-time polymerase chain reaction (qRT-PCR)

To conduct qRT-PCR, total RNA from colonic tissue was isolated using the triazol (Ambion Life Technology, Foster City, CA) and quantified by the NanoDrop technique using the SPARK 10 M (TECAN, Untersbergstrasse, Grodig, Austria). Then, cDNA was constructed using the GoScript™ reverse transcription system (Promega, Madison, WI). RT-PCR was conducted using the SYBER green to check the mRNA expression of TNF-α and IFN-γ in the colon samples. The primer sequences used in this study are listed in Table 1.

**Table 1:** Primer sequences

Target gene	Primer	Nucleotide sequence
GAPDH	F	5’-ACCACAGTCCATGCCATCAC-3’
	R	5’-TCCACCACCCTGTTGCTGTA-3’
TNF-α	F	5’-TAGCCAGGAGGAGAACAGAAAC-3’
	R	5’-CCAGTGAGTGAAAGGGACAGAAC-3’
IFN-γ	F	5’-CAAGTGGCATAGATGTGGAAG-3’
	R	5’-GGCAATACTCATGAATGCATCC-3’

### Statistical analysis

Statistical analysis was performed using the GraphPad Prism 5 software (GraphPad Software, Inc., La Jolla, CA, USA). Statistical values were calculated using a one-way analysis of variance or unpaired *t*-tests. Differences with *p*-values of less than 0.05 were considered statistically significant.

## Results

### Preparation and characterization of FK506-loaded microspheres

**Fig. 1:**
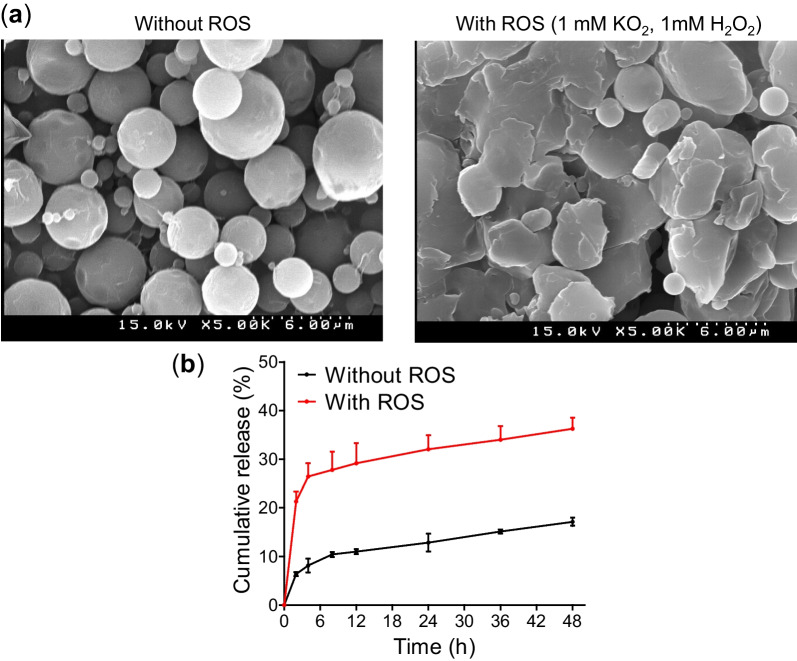
Characterization of TKMs. **a** SEM images of FK506-TKMs without and with ROS treatment (1 mM of H_2_O_2_ and 1 mM of KO_2_) for 48 h. **b** Release profile of FK506. For the release study, 0.2% Tween 20 in PBS (pH: 7.5) with or without 1 mM of H_2_O_2_ and 1 mM of KO_2_ was used as release media

FK506-TKMs showed a uniform and spherical shape under SEM examination. FK506-TKMs showed ROS-dependent degradation when incubated with 1 mM H_2_O_2_ and KO_2_ for 48 h (Fig. 1a). Moreover, in vitro release study in the presence or absence of 1 mM H_2_O_2_ and KO_2_ suggested that ROS-triggered release of FK506 from FK506-TKMs. The presence of ROS (1 mM H_2_O_2_ and KO_2_) in the release media accelerated the release of the FK506 from FK506-TKMs (Fig. 1b). This suggests that the ROS-responsive behavior of FK506-TKMs provided on-demand availability of the payload at the site of inflammation (region with higher levels of ROS) compared to that of the normal tissues. Higher accumulation of drugs in the disease site ensures effectiveness while decreasing the symptoms of systemic adverse effects.

### Effective alleviation of DSS-induced murine colitis by a combination of FK506-TKMs and AMD

**Fig. 2:**
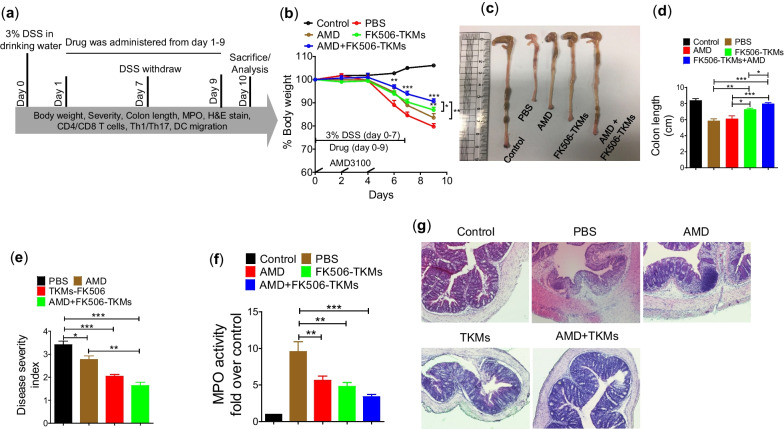
Synergistic effects of AMD and FK506-TKMs on UC treatment. **a** Schematic representation of study timeline. **b** Body weight change of mice in control, PBS, AMD, FK506-TKMs, and AMD + FK506-TKMs treated groups. The values represent mean ± SEM (*n* = 7). **c** Representative image of the colon. **d** Quantitative estimation of colon length. The data represented the mean ± SEM (*n* = 6) **e** Disease severity index. The data represents mean ± SEM (n = 7). **f** MPO activity in the colon. The data is represented as mean ± SEM (*n* = 7). **g** H&E staining of the colon. The mean score of H&E staining was calculated based on the extent of immune cell infiltration, goblet cell loss, and epithelial damage (*n* = 5). *p < 0.05, **p < 0.01, and ***p < 0.001. (Colon lengths of individual mice are presented in supplementary Fig. 1)

Figure 2a shows the schematic representation of the study design. The mice with DSS treatment showed marked loss of body weight. Treatment of AMD or FK506 alone did not prevent weight loss; however, the combination of AMD and FK506-TKMs significantly inhibited weight loss in the mice (Fig. 2b). DSS treatment led to shortening of colon and this was recovered to a maximal extent in the combination group which further supports a synergistic effect of AMD and FK506-TKMs (Fig. 2c, d). Figure 2e shows disease severity index, which was calculated by considering body weight, presence of blood in stool, rectal bleeding, and stool consistency. This value was minimal in the AMD + FK506-TKMs group (*p* < 0.001 vs PBS group). Neutrophil infiltration in colon was increased with DSS treatment. Maximum inhibition of neutrophil infiltration was observed in mice which received a combination therapy of AMD and FK506-TKMs (Fig. 2f). Furthermore, under H&E examination, we observed a maximal damage of colonic epithelium when mice were treated with DSS only. Although AMD and FK506 alone treatments slightly improved the colon morphology, this damage was markedly attenuated when the mice were administered with AMD and FK506-TKMs (Fig. 2g).

### Inhibition of immune cell infiltration and inflammation in *colon* by combination therapy of FK506-TKMs and AMD

**Fig. 3:**
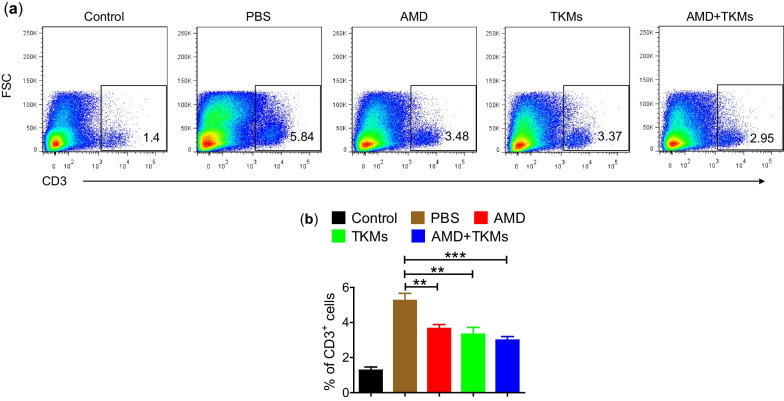
Inhibition of immune cell infiltration in the colon. **a** Representative image of flow cytometric analysis representing the percentage of CD3^+^ cells in control, PBS, AMD, FK506-TKMs, and AMD + FK506-TKMs treated groups. **b** Percentage of CD3^+^ T cells in the colon. The data represents mean ± SEM (n = 5). **p < 0.01, and ***p < 0.001

**Fig. 4:**
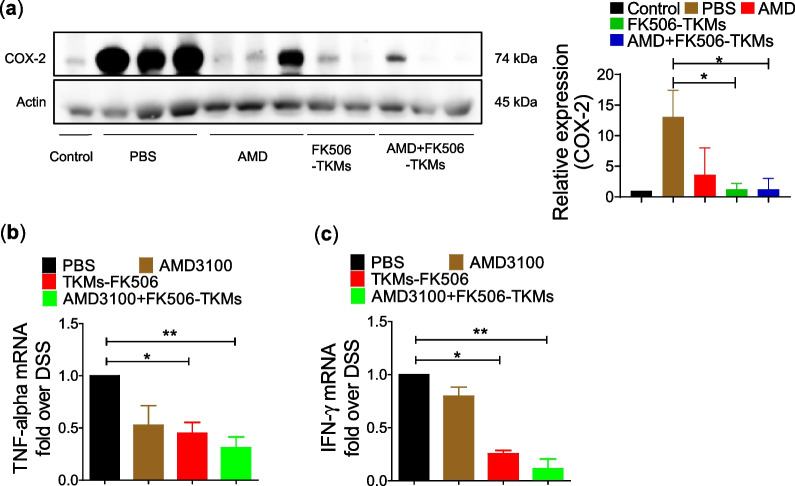
Inhibition of inflammatory protein and genes expression in the colon. **a** Protein expression of COX-2 in the colon **b** mRNA expression of TNF-α in the colon of PBS, AMD, FK506-TKMs, and AMD + FK506-TKMs treated groups. **c** mRNA expression of IFN-γ in the colon of PBS, AMD, FK506-TKMs, and AMD + FK506-TKMs treated groups. The data represents mean ± SEM (n = 4). *p < 0.05, **p < 0.01. (Full western blot data are presented in supplementary Fig. 2)

To investigate the effect of the combinatorial approach of stem cell mobilization and local immunosuppression on immune cell infiltration in the colon, we estimated the percentage of CD3^+^ cells in colonic lamina propria. DSS-treatment significantly increased infiltration of CD3^+^ cells in the colon of PBS treated group (*p* < 0.001) compared to the control. However, the treatment with subcutaneous delivery of AMD, oral delivery of FK506-TKMs, and a combination of AMD + FK506-TKMs (AMD: *p* < 0.01 *vs.* PBS group; FK506-TKMs: *p* < 0.01 *vs.* PBS group; AMD + FK506-TKMs:* p* < 0.001 *vs.* PBS group) significantly inhibited the infiltration of CD3 cells compared to PBS-treated group (Fig. 3a, b). Moreover, to evaluate the inflammation in the colon, inflammatory proteins and genes were evaluated in the colon. The PBS-treated group showed markedly higher expression of COX-2, suggesting the aggravation of inflammation in the colon. The treatment of AMD, FK506-TKMs, and AMD + FK506-TKMs attenuate the expression of COX-2 where the AMD + FK506-TKMs treated group showed the lowest expression of COX-2 (Fig. 4a). This suggested the effectiveness of combinational therapy in inhibiting colonic inflammation. Moreover, a significant decrease in TNF-α and IFN-γ was observed in FK506-TKMs (*p* < 0.05 *vs.* PBS group) and AMD + FK506-TKMs (*p* < 0.01 *vs.* PBS group) compared to PBS treated group (Fig. 4b, c).

### Inhibition of Th1 and Th17 differentiation of CD4^+^ T cells

**Fig. 5:**
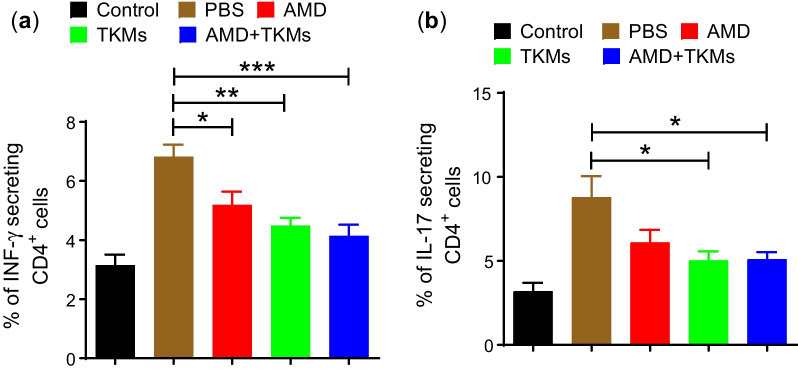
Inhibition of Th1/Th17 differentiation of CD4 T cells. **a** The percentage of IFN^+^CD4^+^ T cells in cMLN determined by FACS. **b** The percentage of IL 17^+^CD4^+^ T cells in cMLN determined by FACS. Data represents ± SEM (n = 4). *p < 0.05, **p < 0.01, and ***p < 0.001

The inflammation in the colon is followed by the activation of the T cells triggering the differentiation of naïve T cells to Th1 or Th17 type pro-inflammatory cells in the colon draining mesenteric lymph nodes. To evaluate the effect on Th1/Th17 differentiation in lymphocytes, ionomycin/PMA re-stimulated lymphocytes were evaluated for INF-γ and IL-17 secreting cells in CD4 cells. In the PBS treated group, higher activation of CD4 cells to INF-γ secreting Th1 cells was observed, which was significantly decreased in the TKMs and AMD + TKMs group (Fig. 5a, b).

### Stem cell migration from bone marrow *to colon*

**Fig. 6:**
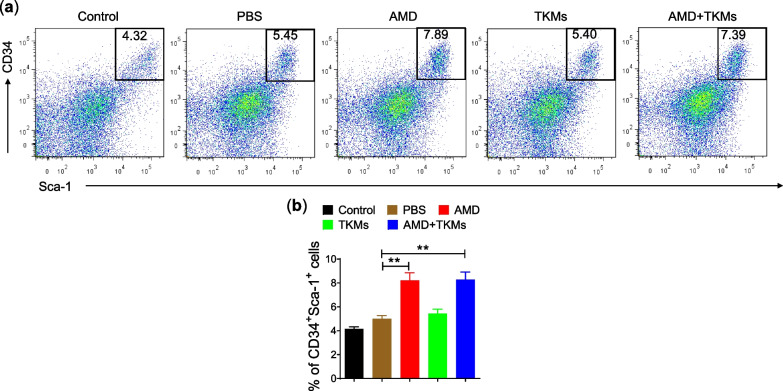
AMD enhanced the stem cell migration to colon. **a** Representative FACS figure showing the percentage of CD34^+^Sca-1^+^ **b** Percentage of these stem cells migrated from bone marrow to colon. Data represents ± SEM (n = 4). *p < 0.05 and ***p < 0.001

Stem cells have an immunomodulatory effect of suppressing inflammation and the regenerative effect for tissue remodeling and repairment. However, stem cells migrate towards the injury by sensing the cytokines produced at the infected sites. This study evaluated the effect of mobilization of endogenous stem cells combined with immunosuppressive drugs in the infected colon. In PBS and TKMs treated groups, higher hematopoietic stem cells (HSCs) (Sca-1^+^CD34^+^ cells) migrated from bone marrow than untreated control mice. However, in AMD and AMD + TKMs treated groups, there was a significantly higher accumulation of HSCs compared to the PBS treated group (Fig. 6a, b). This suggests that AMD effectively mobilizes the HSCs from bone marrow to the inflamed colon.

## Discussion

Stem cell therapy is a promising alternative for treating a variety of diseases. However, the exogenously transferred stem cells undergo apoptosis and senescence; hence, affecting MSC-based therapy. Therefore, there is a need to mobilize endogenous stem cells from bone marrow. This study used a combination of stem cells mobilizer and low-dose FK506 using ROS-responsive particles. From the results, we confirmed that the local delivery of FK506 and stem cell mobilization led to a significant improvement in the therapeutic outcomes in UC. In our previous study, the local delivery of FK506 using newly synthesized ROS responsive polymer improved the therapeutic effect compared to free-FK506. Despite the inhibition of immune cell infiltration into the colon, reduction of body weight during colitis was not prevented with the use of the FK506 microspheres alone. Therefore, additional interventions are needed to prevent bodyweight reduction. To prevent immune cell activation and maintain normal body weight, we used the FK506-TKMs in combination with the CXCR4 antagonist.

AMD (Plerixafor or Mozobil) is a CXCR4 antagonist and is an FDA-approved drug for stem cell mobilization from bone marrow to peripheral blood. CXCR4 is a chemokine receptor highly expressed in HSC. CXCR4 signaling is involved in the retention of HSC in the bone marrow. In contrast, the blockage of CXCR4 resulted in the mobilization of HSC from bone marrow to the peripheral bloodstream. Due to the activity of AMD in mobilizing HSCs from bone marrow to the infected sites, it has been applied for the treatment of several tissue injuries. For example, single-dose AMD was effective in functional recovery of myocardial infractions through neovascularization [27], and the topical application of AMD resulted in the recovery of wounds in diabetic mice [28].

Furthermore, the mobilized HSCs inhibit inflammation through the high expression of costimulatory PD-L1 resulting in inhibition of immune cells [29]. On the other hand, enhancement of angiogenesis and vasculogenesis was associated with the tissue repairing effect of AMD [27, 28]. In colitis, AMD modulated the colonic claudin expressions and improved intestinal barrier function [30].

Mature lymphocytes, monocytes, and neutrophils are affected by CXCR4 signaling. Their migration, homing, and retention is influenced by the chemokine receptors, including CXCR4. Several researchers have reported that the mobilization of the immune cells from bone marrow to the blood and lymph organs or spleen also occurs by using AMD [31]. The effect of AMD was enhanced in several studies when used in combination with low-dose immunosuppressive drugs [20, 29, 32–34]. The use of FK506 in combination with AMD enhances the mobilization of stem cells through increasing SDF-1 production and assists in the migration of stem cells to the inflammation sites [20]. The low-dose FK506 pulls the stem cells from bone marrow niches where SDF-1 (induced by FK506) acts as a strong pulling agent for the circulating stem cells to the injured tissue resulting in an increased accumulation of stem cells at the injured tissue promoting rapid repairment. In our study, we used a very low dose of FK506 to target the site of inflammation using ROS responsive microspheres. This prevents systemic toxicity of FK506 while exerting a potent immunosuppressive activity locally at the inflamed colon. Therefore, in the combination treatment group, the overall stem cell mobilization was found to be similar to the AMD only group in our study. The combination of AMD and targeted FK506 delivery enhanced therapeutics effect in colitis compared to FK506-TKMs or AMD only by increasing stem cell recruitment into the colon and decreasing the immune recruitment or activation locally at the inflammation site.

## Conclusion

This study reported a synergistic drug therapy to alleviate DSS-induced experimental colitis effectively. Locally delivered calcineurin inhibitor and HSC mobilizing agent prevented infiltration of immune cells and colitis progression in DSS-fed mice. Furthermore, FK506-TKMs showed improved effects in colitis compared to free FK506 through local immunosuppression. Furthermore, the immobilized HSCs led to the migration of stem cells into the colon and the acceleration of tissue regeneration through immunomodulation and epithelial regeneration. Therefore, the combined therapeutic regimen proposed might be a milestone in treating colitis.

### Supplementary Information

Additional file 1. 

## Data Availability

Not applicable.

## Abbreviations

UC: Ulcerative colitis
Anti-TNF-α: Anti-tumor necrosis factor- α
GI tract: Gastrointestinal tract
ROS: Reactive oxygen species
IBD: Inflammatory bowel disease
MSCs: Mesenchymal stem cells
FDA: Food and drug administration
HSCs: Hematopoietic stem cells
CXCR4: C-X-C chemokine receptor type 4
TKMs: Thioketal microspheres
AMD: AMD-3100
HPLC: High performance liquid chromatography
SEM: Scanning electron microscope
DSS: Dextran-sulfate sodium
MPO: Myeloperoxidase
H & E: Hematoxylin and Eosin
c-MLN: Colon-draining mesenteric lymph nodes
PMA: Phorbol 12-myristate 13-acetate
FACS: Fluorescence-activated cell sorting
SDS-PAGE: Sodium dodecyl sulfate–polyacrylamide gel electrophroresis
COX-2: Cyclooxygenase-2
HRP: Horseradish peroxidase
IFN-γ: Interferon- γ
SDF-1: Stromal cell-derived factor 1
