# Supplementary material for: Endogenous stem cell mobilization and localized immunosuppression synergistically ameliorate DSS-induced Colitis in mice
Source: Stem Cell Res Ther. 2024 Jun 13;15:167. doi: 10.1186/s13287-024-03777-2 (PMC11170870; doi:10.1186/s13287-024-03777-2)
Supplement: Supplementary file 1 — Additional file 1. [file 13287_2024_3777_MOESM1_ESM.docx]

**Supplementary File**

**Endogenous Stem Cell Mobilization and Localized Immunosuppression Synergistically Ameliorate DSS-induced Colitis in Mice**

Shobha Regmi^1,2^, Shiva Pathak^1,3^, Dinesh Chaudhary^4^, Jong Oh Kim^1^, Joo-Won Nam^1^, Hyung-Sik Kim^5,6^, Hu-Lin Jiang^7,8,9,10^, Dongryeol Ryu^11^, Jong-Hyuk Sung^12,13*^, Simmyung Yook^14,15*^, and Jee-Heon Jeong^4*^


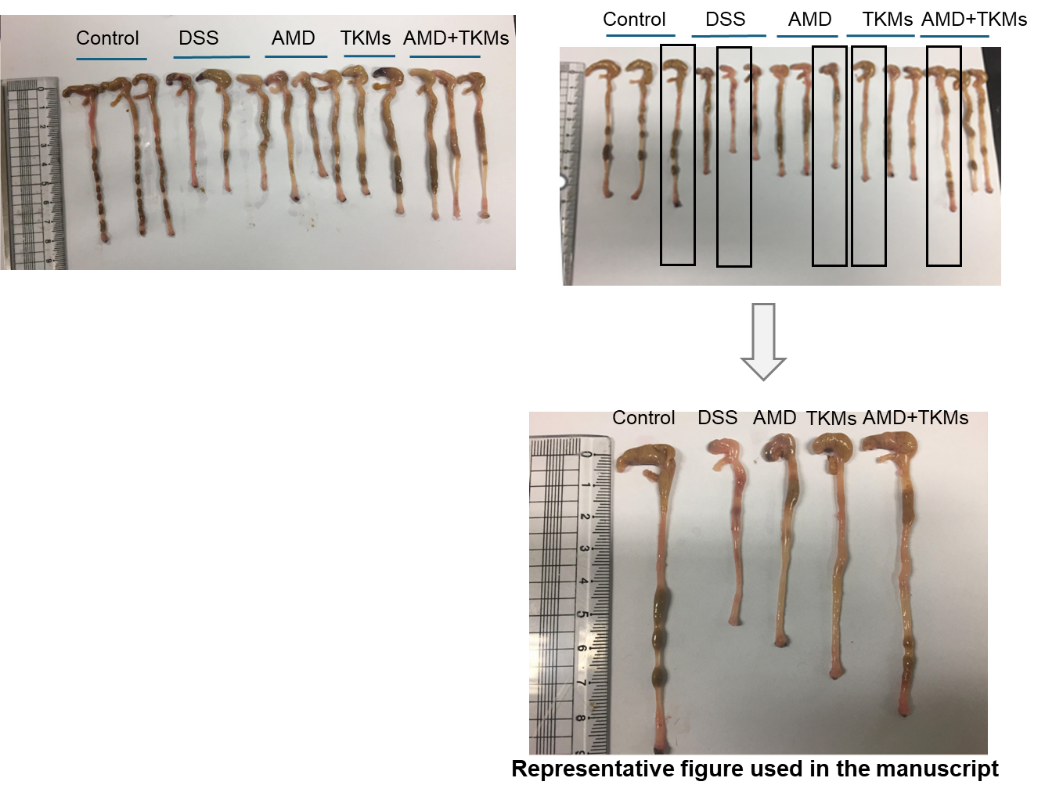


**Supplementary Figure 1**. **Colon length of individual mice from *in vivo* colitis experiments**. Animal experiments were conducted two times.

**Exposure Time-1**


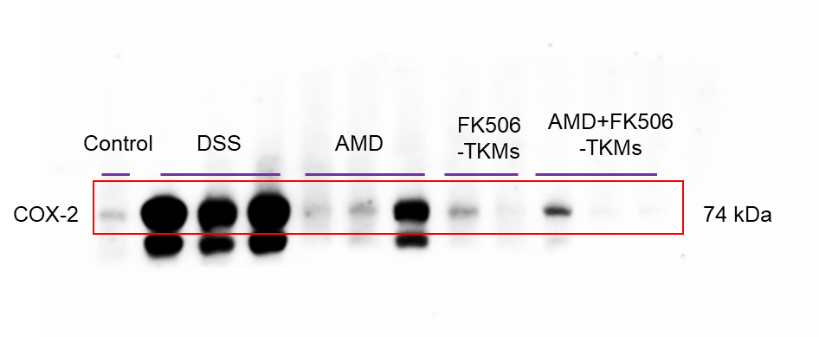

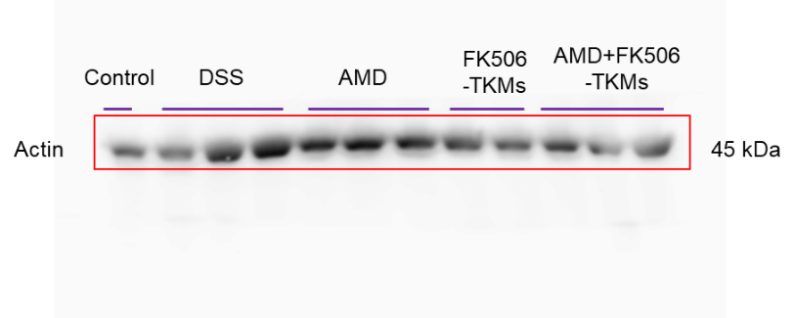


**Exposure Time-2**


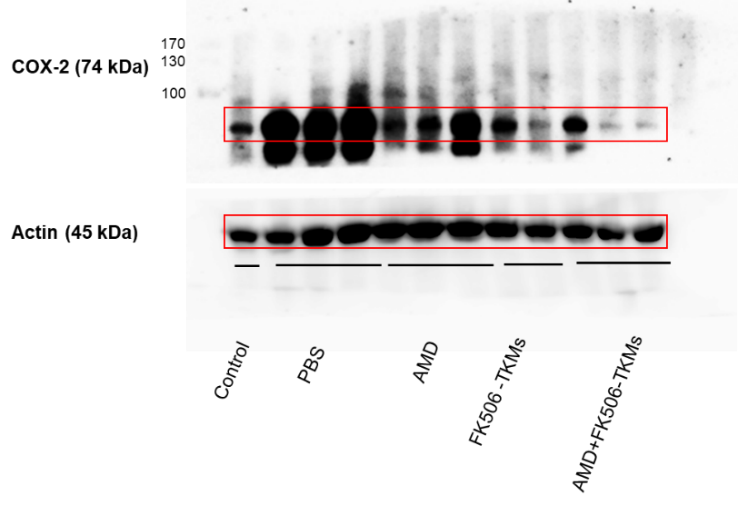


**Supplementary Figure 2**. **Western blot results from the same gel (different exposure time) for representing protein ladder**. Protein-transferred membrane was divided into two parts from 55 kDa (based on the protein ladder, Thermo Scientific^TM^ PageRuler^TM^ prestained protein ladder (10-180 kDa)). The upper portion was used for the detection of COX-2 protein (74 kDa; 1:1000; Cell Signaling Technology; #12282S) while the lower portion was used for actin protein (45 kDa; 1:1000; Cell Signaling Technology; #4970S). Western blot was performed only one time. Moreover, protein ladder was only detected in upper portion but not in lower portion (cut for actin). Here, actin protein was taken as loading control.
